# Transcriptome profiling of two rice genotypes under mild field drought stress during grain-filling stage

**DOI:** 10.1093/aobpla/plab043

**Published:** 2021-07-05

**Authors:** Yuya Liang, Rodante E Tabien, Lee Tarpley, Abdul R Mohammed, Endang M Septiningsih

**Affiliations:** 1 Department of Soil and Crop Sciences, Texas A&M University, College Station, TX 77843, USA; 2 Texas A&M Agrilife Research Center, Beaumont, TX 77713, USA

**Keywords:** Differentially expressed genes (DEGs), drought, grain-filling stage, rice (*Oryza sativa*), RNA-seq

## Abstract

Drought is one of the most critical abiotic stresses that threaten crop production worldwide. This stress affects the rice crop in all stages of rice development; however, the occurrence during reproductive and grain-filling stages has the most impact on grain yield. Although many global transcriptomic studies have been performed during the reproductive stage in rice, very limited information is available for the grain-filling stage. Hence, we intend to investigate how the rice plant responds to drought stress during the grain-filling stage and how the responses change over time under field conditions. Two rice genotypes were selected for RNA-seq analysis: ‘4610’, previously reported as a moderately tolerant breeding line, and Rondo, an elite *indica* rice cultivar susceptible to drought conditions. Additionally, 10 agronomic traits were evaluated under normal irrigated and drought conditions. Leaf tissues were collected during grain-filling stages at two time points, 14 and 21 days after the drought treatment, from both the drought field and normal irrigated field conditions. Based on agronomic performances, ‘4610’ was less negatively affected than Rondo under mild drought conditions, and expression profiling largely aligned with the phenotypic data. The transcriptomic data indicated that, in general, ‘4610’ had much earlier responses than its counterpart in mitigating the impact of drought stress. Several key genes and gene families related to drought stress or stress-related conditions were found differentially expressed in this study, including transcription factors, drought tolerance genes and reactive oxygen species scavengers. Furthermore, this study identified novel differentially expressed genes (DEGs) without function annotations that may play roles in drought tolerance-related functions. Some of the important DEGs detected in this study can be targeted for future research.

## Teaser

Few global transcriptomic studies have been performed in rice to study drought stress at the grain-filling stage. This study investigated drought stress responses of two rice genotypes using RNA-seq analysis at the grain-filling stage under drought and normal irrigated conditions. The transcriptomic data indicated that the moderately tolerant cultivar had significantly earlier responses than the susceptible cultivar in mitigating the impact of drought stress. Several key gene families were found differentially expressed, including transcription factors, drought tolerance genes and reactive oxygen species scavengers. This study also identified novel unannotated differentially expressed genes that can be investigated further.

## Introduction

Rice (*Oryza sativa* L.) is a staple food that feeds about 3.5 billion people, more than half of the world’s population (Ricepedia 2013). The global population is projected to reach 9.1 billion by 2050 (Food and Agriculture Organization of the United Nations 2009); therefore, an increase in global rice production is critical to keep pace with the rising global demand for food. However, rice production faces daunting challenges, including abiotic stress problems. Drought is one of the main obstacles to rice production; nearly 50 % of rice farming is affected worldwide (Bouman *et al.* 2005). This stress can cause limited nutrient uptake, cell dehydration and the production of excessive metabolic compounds of reactive oxygen species (ROS) (Price *et al.* 1989; Choudhury *et al.* 2017). Simple cell dehydration may damage cells, including membrane dysfunction, misfolded protein and cytoskeletal damage. Likewise, ROS may cause DNA, protein and membrane damages (Mittler 2002; Tenhaken 2015).

Drought tolerance mechanisms are complex. In legumes, although drought ultimately affects the productivity of grains at all growth stages, the incidence during reproductive and grain development stages results in more substantial grain production loss (Farooq *et al.* 2017). A similar phenomenon was also observed in cereals, such as wheat and rice (Tripathy *et al.* 2000; Farooq *et al.* 2014; Prathap *et al.* 2019). Drought stress during the reproductive stage can reduce yield dramatically (Garrity and O’Toole 1994; Venuprasad *et al.* 2007; Raman *et al.* 2012). Likewise, many studies have shown that drought stress also has significant impacts during the grain-filling stage, including decreased photosystem II (PSII) activity (Pieters and El Souki 2005), rapid accumulation of free radical (O_2_^·−^) and hydrogen peroxide (H_2_O_2_) (Wang *et al.* 2010), fewer sink activities and smaller sink sizes (Dong *et al.* 2014) and early senescence leading to decreased starch in matured grains and the loose packaging of the starch granules (Prathap *et al.* 2019).

There are two main categories of drought tolerance mechanisms, preventing water loss and preventing damage caused by dehydration (McDowell *et al.* 2008; Farooq *et al.* 2009). Abscisic acid (ABA) regulation (Munemasa *et al.* 2015), K^+^ concentration and membrane potential (Corratge-Faillie *et al.* 2017) can regulate stomatal conductance to reduce water loss (Chaves *et al.* 2008). Overexpression of drought-induced genes such as calcium-dependent protein kinase (CDPK), NAM, ATAF and CUC (NAC) transcription factors (TFs), and dehydration-responsive element-binding proteins (DREBs) contribute to the drought tolerance response in rice (Saijo *et al.* 2000; Hu *et al.* 2006; Chen *et al.* 2008).

Rice transcriptomic studies under drought conditions in different scenarios have been reported, including during seedling stage and reproductive stage (Lenka *et al.* 2011; Huang *et al.* 2014; Plessis *et al.* 2015; Wilkins *et al.* 2016; Zhang *et al.* 2016; Yoo *et al.* 2017). To our knowledge, however, transcriptome profiling of drought response mechanisms during grain-filling stage is very limited. Hence, our current study aimed to investigate the molecular mechanisms underlying drought tolerance during the grain-filling stage and to evaluate how the responses change during this period under realistic field conditions. A time series of RNA-seq using two rice genotypes having different responses to drought, i.e. Rondo and ‘4610’, was performed. Additionally, some agronomic traits were measured. Our results showed that, in general, earlier cellular events, signalling cascades and stress-responsive mechanisms in ‘4610’ than Rondo were observed. Overall, both transcriptome profiling and phenotypic data concurred that ‘4610’ was more tolerant under drought stress. Some of the differentially expressed genes (DEGs) detected in this study can be further investigated for further molecular studies and manipulation for crop improvement to enhance plant survival and mitigate grain yield reduction under drought conditions.

## Materials and Methods

### Plant materials and drought treatment

Two rice genotypes with different performances under drought stress, Rondo and ‘4610’, were selected for this study. Rondo is an elite *indica* long-grain rice cultivar, developed by the USDA-ARS through mutation breeding from the Chinese *indica* germplasm ‘4484’ (PI 615022). This cultivar has a high yield, is resistant to various diseases (Yan and McClung 2010) and is mainly grown in the mid-south rice belt of the USA, including Arkansas, Louisiana, Mississippi, Missouri and Texas. Rondo is also being planted for commercial organic rice production in Texas. However, this cultivar is susceptible to drought stress (Tabien *et al.* 2015; Dou *et al.* 2016). On the other hand, ‘4610’ is an *indica* long-grain (PI615037) introduced from China (Dilday *et al.* 2001), previously selected from herbicide-resistant screening at the Texas A&M AgriLife Research Center in Beaumont, TX, USA, and was reported as less affected by drought (Tabien *et al.* 2015). It is intriguing to understand how these two different cultivars respond to field drought stress and how to use this information to enhance the tolerance of the elite cultivar Rondo and possibly other susceptible *indica* cultivars or breeding lines in the future.

A field experiment was performed at the Texas A&M AgriLife Beaumont Research Center in 2016 and 2017. Beaumont is located in East Texas and has an average of 18–31.6 °C daytime temperature and 13.72 cm precipitation per month during June–October, the duration of this experiment. Unfortunately, our experiment in 2017 was affected by the tropical storm Harvey. Considering that Rondo and 4610 had previously been screened under drought conditions at the same experimental station (Tabien *et al.* 2015; Dou *et al.* 2016), we envision that our data can be used as further phenotypic confirmation of previous screening results.

The two rice genotypes were directly sowed in the experimental rice field using a randomized complete block design (RCBD) with three replications in normal irrigated and drought conditions. The rates and timing of fertilizer and herbicide were similar for both fields. A total of 56 kg P per hectare (as P_2_O_5_) was applied during pre-season, and 224 kg ha^–1^ N was applied as urea as follows: 56 pre-plant, 90 at the permanent flood and 78 at panicle differentiation. Glyphosate was applied pre-season; clomazone + halosulfuron-methyl post-plant, and cyhalofop at the permanent flood and 14 days later. The heavy clay soil was representative of rice farms in the area. All replicates were planted in three-row blocks; each block has 6 m in length and 18 cm between rows. Drought treatment was applied once >50 % of plants had flowered by draining the field completely. There was a light rain a few days after the draining; however, we were able to manage and maintain the drought conditions afterward and performed the tissue sampling for RNA-seq analysis on the 14th day and 21st day after the draining. The drought field was then rewatered right after the second tissue sampling.

### Phenotypic evaluation

A total of 10 agronomic traits were measured at physiological maturity; these were filled grain number per panicle, unfilled grain number per panicle, spikelet fertility, filled grain weight per plot, unfilled grain weight per plot, total grain weight per plot, plant dry weight, hundred-seed weight, panicle length and yield. The 10 representative panicles from each plot were used to count the grain number, including filled and unfilled grain numbers. Spikelet fertility was calculated from filled and unfilled grain numbers on each of the selected panicles. All panicles in each plot were manually threshed and manually separated as filled and unfilled grains. Filled and unfilled grain data were then used to calculate filled grain weight, unfilled grain weight and total grain weight per plot. Plant dry weight was measured for the above-ground part of the plant after air-drying for 7 days according to the protocol of the Beaumont Station Rice Breeding Lab. Hundred-seed weight was the average of three subsamples of 100 randomly selected filled grains. Panicle length was measured as average from 20 panicles in each plot. The yield was counted from the total grain weight per plot. The student’s *t*-test was performed using JMP Pro 12.2 to determine the significance of difference for all traits.

### RNA extraction and sequencing

RNA was extracted from leaf tissues collected at two different time points, 14 and 21 days after the field for the drought treatment was drained. These time points corresponded to the earlier and later grain-filling stages, respectively. Early symptoms of drought (leaf rolling) were observed during the samplings at both time points. It was also observed that there were no drastic differences in the severity of drought across both time points; the level of drought stress at both time points was mild. Leaf tissue samples were also taken from the normal irrigated field at the same time points. The samplings were performed in the morning, around 10–11:30 am. For each condition and each time point, three biological replicates were collected. These replicates were from three different plants, and the leaves collected on the 14th day and the 21st day were from the same plant. The second leaves, counted from the top, on the main stem were collected on the 14th day. The third leaves, counted from the top, on the main stem were collected on the 21st day. RNA was extracted using TRIzol reagent, followed by QIAgen RNeasy Plant Mini Kit. The samples were not DNAse-treated; however, based on the assessment on agarose gel, there was no DNA contamination in any of the RNA samples. RNA quality was determined by 28S to 18S ribosomal RNA ratio and samples with a ratio within the range of 1.8–2.2 were checked further using Agilent 2100 Bioanalyzer (Santa Clara, CA, USA). The samples that passed the quality check were used for the library preparation. TruSeq Stranded RNA-seq libraries were prepped at Texas A&M AgriLife Genomics and Bioinformatics Service (TxGen; College Station, TX, USA) as per Standard Operating Protocol (Illumina). The libraries were run on multiple lanes of an Illumina HiSeq 4000 (San Diego, CA, USA) to provide at least 25 million reads (75 nt pair-end) per sample.

### Data processing

In total, 12 cDNA libraries were made for sequencing, including three biological replicates for each treatment. Trimmomatic version 0.36 (Bolger *et al.* 2014) PE –Phred 33 command was used for quality control using the following steps: (i) raw sequencing reads were trimmed to remove adaptors; (ii) low-quality bases with a quality score less than 20 on the ends and tails of reads were removed; (iii) reads were scanned with a 5-bp sliding window and were removed when the average quality per bp dropped below 20; (iv) reads below the 25 bases long were dropped; (v) reads without correspondence read pairs were dropped. *Oryza sativa* spp. *japonica* genome Nipponbare (International Rice Genome Sequencing Project (IRGSP)-1.0; Sasaki and Burr 2000; Kawahara *et al.* 2013) was used as a reference in this study. The rice reference sequence and gene annotation files were downloaded from EnsemblPlants (http://plants.ensembl.org). HISAT2 version 2.1.0 (Kim *et al.* 2015) was used to align the reads to the reference genome sequence. Thereafter, StringTie v1.3.4d (Pertea *et al.* 2015) was used to assemble the transcripts within the regions and obtain the gene counts.

### Differential expression and gene ontology enrichment analyses

Differential expression analysis was performed in R studio using DESeq2 v1.26 (Love *et al.* 2014) with reads normalizing and variance stabilizing transformation to account for library size and sequencing depth differences. A generalized linear model of $Y=\tau_{Geno}+\gamma_{Trt}+\tau_{Geno}\times \gamma_{Trt}$, where *Y* is the read count for each gene, with three explanatory variables, including genotype, treatment and interaction between genotype and treatment, was used in this study. Due to the different growing stages, data from the two time points were fitted in the model separately. Internally, *P*-values were adjusted for multiple testing using the Benjamini–Hochberg method in the DESeq2 package. Genes with false discovery rate (FDR) adjusted *P*-value (*P*_adj_) < 0.05 were identified as DEGs. There were six DEG lists used for the gene ontology (GO) enrichment analysis in both reproductive and grain-filling stages, including uniquely upregulated and downregulated DEGs in ‘4610’, uniquely upregulated and downregulated DEGs in Rondo, commonly upregulated DEGs in both cultivars and commonly downregulated DEGs in both cultivars. Gene ontology enrichment analysis was performed using the web-based tool PANTHER v15.0 (Mi *et al.* 2019). For GO enrichment analysis, Fisher’s Exact test was used for the main statistical analysis with FDR adjusted by the Benjamini–Hochberg method. Gene ontology terms with FDR < 0.05 were identified as significant.

## Results and Discussion

### Agronomic performance under mild field drought stress

Agronomic characters were collected from 12 plots, covering three replicates for each genotype under each condition. Under the irrigated condition, Rondo had a significantly higher number of both filled and unfilled grains than those of ‘4610’. The panicle length of Rondo was also significantly longer than ‘4610’. On the other hand, ‘4610’ had a significantly higher hundred-seed weight than Rondo (Table 1). Under drought treatment, most agronomic characters declined in both ‘4610’ and Rondo except panicle length and hundred-seed weight of ‘4610’(19.5 cm and 2.9 g in both conditions, respectively). Additionally, ‘4610’ had significantly higher filled grain numbers than Rondo under drought conditions (69.5 and 58.9, respectively), even though under irrigated conditions, the value for this trait was higher in Rondo than ‘4610’. Moreover, ‘4610’ had significantly higher spikelet fertility than Rondo under drought conditions (69.5 and 65.9 %, respectively), while there was no difference in this trait between the two genotypes under irrigated conditions. Although not statistically significant; overall, ‘4610’ had a lower yield reduction under drought than Rondo (7.7 and 12.7 %, respectively). Taken together, under mild drought conditions, ‘4610’ was less affected by drought than Rondo. These data confirmed the previous report (Tabien *et al.* 2015).

**Table 1.** Effect of variety on yield-related traits under irrigated and drought condition.

	Irrigated		Drought	
	‘4610’	Rondo	‘4610’	Rondo
(1) Filled grain number per panicle	68.2*	80.1	69.5*	58.9
(2) Unfilled grain number per panicle	21.2*	27.5	31.2	30.5
(3) Spikelet fertility (%)	76.9	74.3	69.5*	65.9
(4) Filled grain weight (g) per plot	77.3	63.8	55.3	43.7
(5) Unfilled grain weight (g) per plot	5.7	6.9	4.1	4.6
(6) Total grain weight (g) per plot	83.0	70.7	59.5	48.3
(7) Plant dry weight (g)	200.76	163.9	126.6	117.3
(8) Hundred-seed weight (g)	2.9**	2.7	2.9**	2.6
(9) Panicle length (cm)	19.5**	21.6	19.5	19.5
(10) Yield (kg ha^–1^)	10,666	10,351	9,850.04	9,035.18

Traits with ‘*’ and ‘**’ indicates that the difference between the two cultivars under the same condition was significant by *P* < 0.05 and *P* < 0.001, respectively.

### Principal component analysis of RNA-seq samples and the number of DEGs

**Figure 1.**
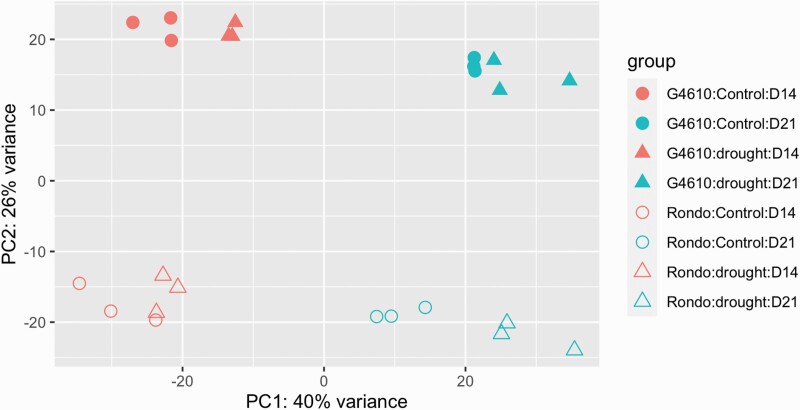
Principal component analysis (PCA) of RNA-seq samples. The colour differences indicate the different time of sampling; the shape differences indicate the irrigated control (circles) and drought treatment (triangles) groups; solid shapes indicate ‘4610’ and hollow shapes indicate Rondo, as depicted in the figure legend on the right.

There were three explanatory variables in the current study, and these were genotype (‘4610’ and Rondo), treatment (irrigated and drought) and time point (14 days and 21 days after drought treatment). To explore the comprehensive gene expression differences of the drought susceptible elite cultivar Rondo and the moderate-tolerant breeding line ‘4610’ under different factors, a total of 38 909 annotated rice genes with at least 10 counts were used for principal component analysis (PCA) as the first step of data exploration. As sources of variations were explored, the different sample collection time points appeared to be the most significant factor in explaining gene expression variations. For the first principal component (PC), which explained 40 % of the total variance (Fig. 1), the sample collection time points showed the dominant loading. The second most important factor appeared to be genotype, which displayed the dominant loading for PC2, which explained 26 % of the total variance. As samples from the same genotype grouped close to each other regardless of the irrigated or drought condition, meaning samples with the same genotype had relatively similar expression patterns.

**Figure 2.**
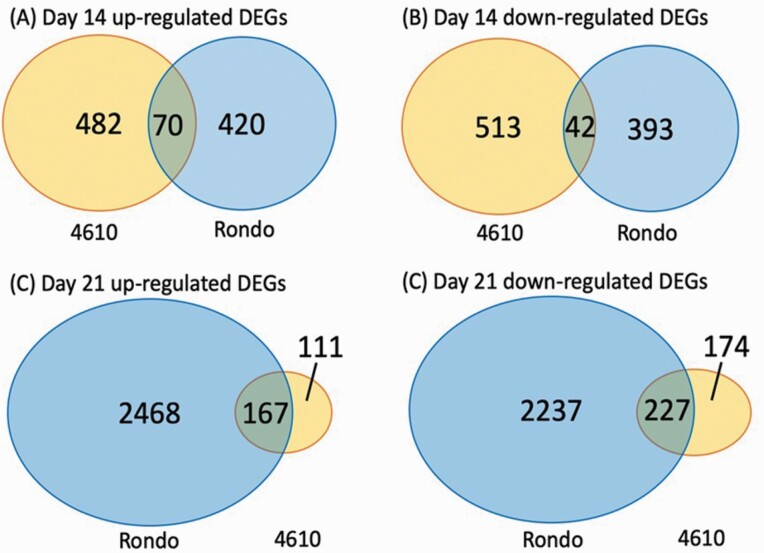
Venn diagrams of DEGs between the moderate-tolerant genotype ‘4610’ and the susceptible genotype Rondo. (A) Upregulated DEGs at 14 days after drought treatment. (B) Downregulated DEGs at 14 days after drought treatment. (C) Upregulated DEGs at 21 days after drought treatment. (D) Downregulated DEGs at 21 days after drought treatment.

To minimize the effect of different time points, data from 14- and 21-day time points were analysed independently. Principal component analysis separated by the two time points suggested that genotype explained 40 % of the total variance on Day 14 and 38 % of the total variance on Day 21 **[see****Supporting Information—Fig. S1****]**. A Venn diagram illustrated the number of DEGs for the four different comparisons (Fig. 2). A total of 1107 and 925 genes were identified to be differentially expressed under drought stress for ‘4610’ and Rondo at 14-day time point with 552 and 490 upregulated genes, and 555 and 435 downregulated genes in ‘4610’ and Rondo, respectively **[see****Supporting Information—Table S1****]**. Among all DEGs, there were 70 upregulated and 42 downregulated DEGs in common (Fig. 2). For the 21-day time point, there were 679 and 5099 DEGs identified, with 278 and 2635 upregulated genes **[see****Supporting Information—Table S2****]** and 401 and 2464 downregulated genes in ‘4610’ and Rondo, respectively. Among all DEGs, there were 167 upregulated and 227 downregulated DEGs commonly identified in both genotypes (Fig. 2).

**Figure 3.**
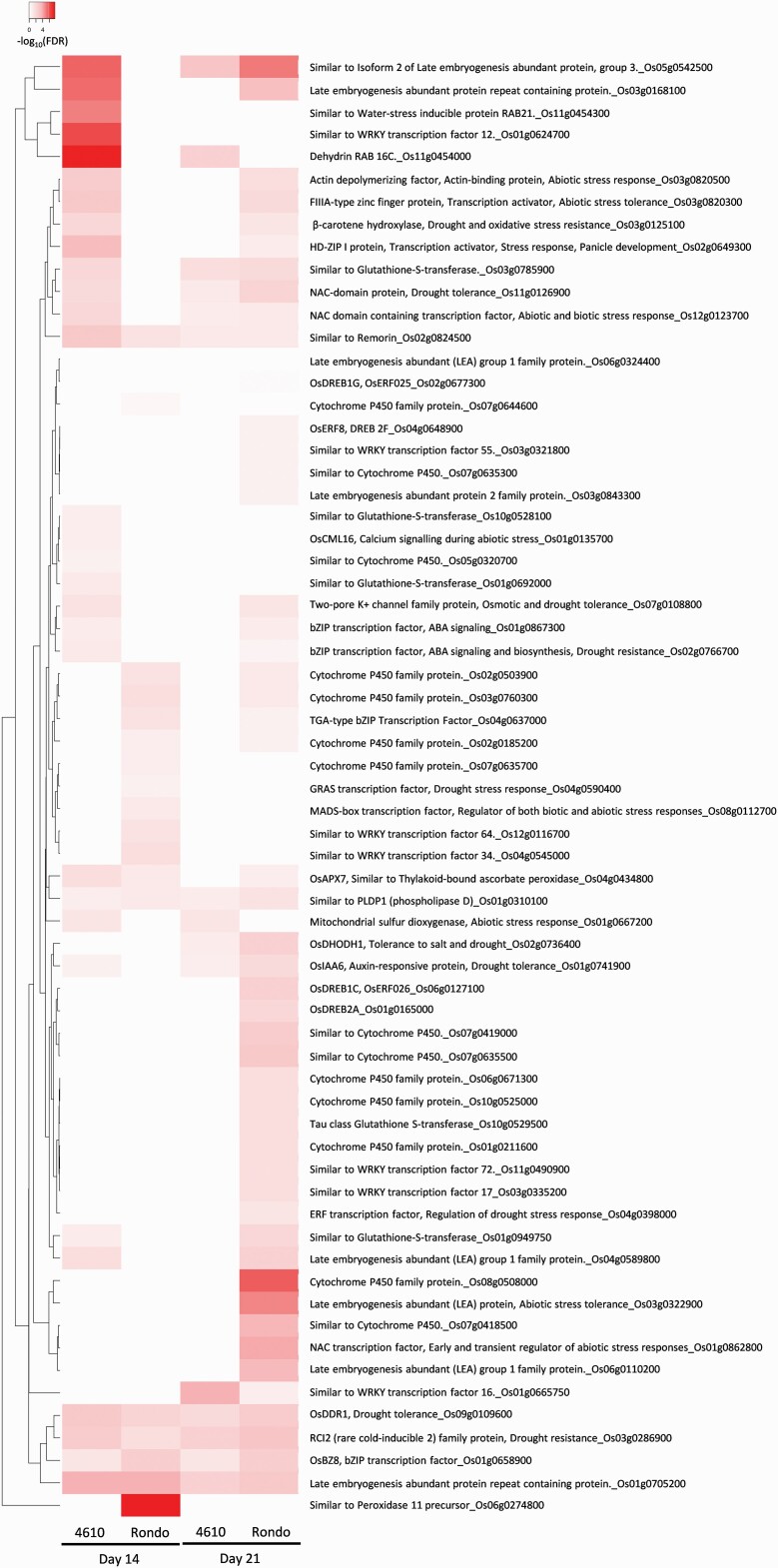
The expression differences of known drought-related DEGs between ‘4610’ and Rondo, in reproductive (Day 14) and grain-filling stages (Day 21), including TFs, cytochrome P450 families, LEA genes, ROS scavengers and other drought-related genes. Heatmap labelled in white indicates log2 fold-change (LFC) equal to 0, meaning the expression level had no difference between control and drought conditions. A higher LFC means the gene had a higher expression level compared to the same gene under control condition.

It was interesting to note that, in general, 4610 had a higher number of DEGs at the earlier stage of grain filling than Rondo; on the other hand, Rondo had a much higher number of DEGs than 4610 on the later stage. This phenomenon suggests that 4610 may give a more rapid response to drought stress than Rondo. However, at the later stage, it seems that Rondo gave an ‘overacting’ response to the stress, which in turn may negatively impact the plant’s cellular homeostasis. This occurrence may also partially explain a better performance in 4610 under drought stress compared to Rondo. A more rapid and stronger drought-responsive regulation in drought-resistant upland rice than a lowland drought susceptible cultivar was also reported in a previous study (Teng *et al.* 2014). A study in barley revealed that a rapid upregulation of dehydrin under dehydration conditions is the a key characteristic of drought-tolerant cultivar (Park *et al.* 2006). Our analysis also showed that at earlier time point dehydrin rab (responsive to ABA) 16C (*Os11g0454000*) and Rab21 (*Os11g454300*) were strongly upregulated in 4610, but not in Rondo (Fig. 3). Here, we were interested in further dissecting the underlying molecular genetic factors that may contribute to the performance of the cultivars under drought conditions.

### Molecular response to drought at the early stage of grain filling (14-day time point)

There were 106 DEGs identified having interaction between genotype and treatment at 14-day time point **[see****Supporting Information—Tables S1****and****S3****]**, including some TFs (*Os12g0515500*, *Os01g0584900*, *Os05g0322900*, *Os04g0656500* and *Os09g0434500*), stress-related genes (the stress upregulated Nod 19 family protein (*Os08g0538600*), a gene related to calcium signalling during abiotic stress (*Os01g0135700*), and a gene contributing to the regulation of abiotic stress responses (*Os07g0129200*) and transporters (Os02g0518600, Os03g0226400 and Os02g0620600). To understand the functional classification underlying these unique DEGs, GO enrichment analysis was performed. There were 12 GO terms enriched in biological process (BP), including response to stimulus (GO:0050896), response to stress (GO:0006950), defence response (GO:0006952) and response to lipid, and 10 GO terms enriched in molecular function (MF), including hormone binding (GO:0042562), abscisic acid binding (GO:0010427) and isoprenoid binding (GO:0019840). However, there were no cellular component (CC) GO terms enriched **[see****Supporting Information—Table S3****]**.

Among 482 uniquely upregulated DEGs in ‘4610’, there were 20 TFs and 17 stress-related genes. Gene ontology analysis further revealed that a total of 76 GO terms were enriched, including 58 BP, 13 MF and 5 CC **[see****Supporting Information—Table S3****]**. Among 76 GO terms, 14 of them were stress-related (Table 2). On the contrary, none of these GO terms were significant in upregulated DEGs in Rondo. Among 420 uniquely upregulated DEGs in Rondo, there were 73 GO terms enriched, including 42 BP, 14 MF and 17 CC **[see****Supporting Information—Table S3****]**. Among 70 commonly upregulated DEGs including one bZIP TF (*Os01g0658900*), five known drought-responsive genes including late embryogenesis abundant (LEA) proteins (*Os01g0705200*, *Os11g0454200*), RIC2 family protein (*Os03g0286900*), ascorbate peroxidase (APX) (*Os04g0434800*) and drought and salt stress response 1 (*Os09g0109600*), and two heat shock proteins (HSP) (*Os02g0232000*, *Os03g0277300*) **[see****Supporting Information—Table S1****]**. There were only six GO terms enriched in CC, including those that play roles in the vacuole and membrane-related functions, such as vacuolar iron transporter (*Os04g0538400*), mitochondrial import inner membrane translocase subunit (*Os03g0305600*) and DDHD domain-containing protein (*Os08g0110700*). Several studies have shown that vacuole and vacuole transporters involve in complex cellular networks and facilitate plants to adjust environmental changes, including salinity tolerance, calcium signalling and cellular pH homeostasis (Martinoia *et al.* 2007). On the other hand, ROS can be generated at the plasma membrane, which may affect membrane stability (Impa *et al.* 2012). In addition, H_2_O_2_ can also cross plant membranes and further activate downstream stress-responsive signalling (Pitzschke *et al.* 2006). However, there was no enrichment on GO terms for BP and MF **[see****Supporting Information—Table S3****]**.

**Table 2.** Stress-related GO categories of ‘4610’ uniquely upregulated DEGs during reproductive stage.

Biological process	GO ID	Number of genes in ‘4610’	FDR	Number of genes in Rondo	FDR
Response to temperature stimulus	GO:0009266	21	5.56E-15	5	ns
Response to abiotic stimulus	GO:0009628	29	5.71E-09	12	ns
Response to heat	GO:0009408	13	9.69E-08	3	ns
Response to osmotic stress	GO:0006970	14	3.13E-07	1	ns
Response to salt stress	GO:0009651	12	4.15E-06	1	ns
Oxidation–reduction process	GO:0055114	50	2.07E-05	37	ns
Response to stimulus	GO:0050896	63	2.32E-04	57	ns
Response to ROS	GO:0000302	8	2.33E-04	2	ns
Response to stress	GO:0006950	36	3.90E-03	23	ns
Response to water deprivation	GO:0009414	7	4.03E-03	3	ns
Response to water	GO:0009415	7	4.96E-03	4	ns
Positive regulation of transcription from RNA polymerase II promoter in response to stress	GO:0036003	4	2.28E-02	2	ns
Glutathione metabolic process	GO:0006749	6	3.15E-02	0	ns
Regulation of DNA-templated transcription in response to stress	GO:0043620	4	3.49E-02	2	ns

Downregulated DEGs were also enriched in distinct categories between ‘4610’ and Rondo. There were 101 BP, 37 MF and 45 CC enriched among 513 uniquely downregulated DEGs in ‘4610’, whereas Rondo had 61 BP, 34 MF and 8 CC among 393 unique DEGs **[see****Supporting Information—Table S3****]**. Interestingly, several photosynthesis-related GO terms were enriched in ‘4610’, including PSII repair (GO:0010206), chlorophyll biosynthetic process (GO:0015995), photosynthesis, light reaction (GO:0019684), photosynthesis (GO:0015979) and chlorophyll metabolic process (GO:0015994). Conversely, most of these photosynthesis-related GO terms were not enriched in Rondo downregulated DEGs, whereas the response to stress (GO:0006950) was enriched with 43 DEGs **[see****Supporting Information—Table S3****]**. Different mechanisms exist through which the rate of photosynthesis under drought stress is suppressed. For example, stomatal closure, diminished CO_2_ influx, decreased Rubisco activity and ROS accumulation (Farooq *et al.* 2009). A strong positive selection on photosynthetic genes conferred upland rice with better drought tolerance than lowland rice, with less photosynthesis rate decline (Zhang *et al.* 2016). The different results might be due to various factors such as plant growth stage and genotype. Many drought transcriptomics studies in rice were conducted during the seedling, vegetative or reproductive stages (Chen *et al.* 2017; Lenka *et al.* 2011; Huang *et al.* 2014; Zhang *et al.* 2016; Borah *et al.* 2017; Tarun *et al.* 2020); however, the responsive mechanisms may differ during the grain-filling stage and need further investigation. A previous study showed that both tolerant and susceptible cultivars had severely decreased photosynthesis rates. The increased abundances of ROS scavengers such as chloroplastic superoxide dismutase and dehydroascorbate reductase, which provide antioxidant protection against damage by dehydration, contributed to tolerance (Ji *et al.* 2012). The two varieties may also have unique adaptation or tolerance mechanisms in response to drought stress. Only 42 DEGs were commonly downregulated in both cultivars with 9 BP and 2 MF terms were significantly enriched. Among the common DEGs, four of them were directly related to yield-component traits such as panicle-branching, grain weight and grain size **[see****Supporting Information—Tables S1****and****S3****]**.

### Molecular response to drought during the later stage of grain filling (21-day time point)

There were 280 DEGs identified having interaction between genotype and treatment, including some TFs, stress-responsive genes and HSPs at 21-day time point **[see****Supporting Information—Table S2****]**. These DEGs were enriched in 27 BP, 47 MF and 17 CC GO terms **[see****Supporting Information—Table S4****]**. The BP terms included a response to H_2_O_2_ (GO:0042542), response to ROS (GO:0000302), chaperone-mediated protein folding (GO:0061077) and protein folding (GO:0006457), and the MF included several membrane activity-related GO terms **[see****Supporting Information—Table S4****]**. Notably, there were chloroplast stroma (GO:0009570), plastid stroma (GO:0009532) and chloroplast (GO:0009507) among CC terms **[see****Supporting Information—Table S4****]**. These indicated ‘4610’ and Rondo had different responses in various functions under drought.

There were 111 upregulated DEGs solely in ‘4610’, including a WRKY TF (*Os01g0665750*), two stress-related genes (*Os01g0667200*, *Os06g0682900*) and five HSPs **[see****Supporting Information—Table S2****]**. These DEGs can be functionally characterized into 15 BP, 4 MF and 1 CC terms **[see****Supporting Information—Table S4****]**. The top five BP GO terms with the largest fold-change were glyoxylate cycle (GO:0006097), glyoxylate metabolic process (GO:0046487), response to heat (GO:0009408), protein folding (GO:0006457) and small molecule catabolic process (GO:0044282) **[see****Supporting Information—Table S4****]**. On the other hand, 2468 uniquely upregulated DEGs in Rondo were enriched in 203 BP, 86 MF and 54 CC terms **[see****Supporting Information—Table S2****and****S4****]**. The top five BP with the largest fold-change were sesquiterpene biosynthetic process (GO:0051762), sesquiterpene metabolic process (GO:0051761), ceramide biosynthetic process (GO:0046513), terpene biosynthetic process (GO:0046246) and acylglycerol metabolic process (GO:0006639). Additionally, three stress-responsive terms were also enriched, including regulation of response to stress (GO:0080134), response to salt stress (GO:0009651), response to osmotic stress (GO:0006970), response to stress (GO:0006950), response to water deprivation (GO:0009414) and response to water (GO:0009415) **[see****Supporting Information—Table S3****]**.

There were also 167 DEGs that were commonly upregulated between ‘4610’ and Rondo, including bZIP TF (*Os01g0658900*), LEA protein (*Os01g0705200*), auxin-responsive protein (*Os01g0741900*), RIC2 protein (*Os03g0286900*), glutathione-S-transferase (GST) (*Os03g0785900*), NAC-domain proteins (*Os11g0126900*, *Os12g0123700*) and a small peptide that regulate drought tolerance (*Os09g0109600*) **[see****Supporting Information—Table S2****]**. And the 167 DEGs can be characterized in 42 BP and 7 MF terms, including glycerophospholipid catabolic process (GO:0046475), chlorophyll catabolic process (GO:0015996) and fatty acid beta-oxidation using acyl-CoA oxidase (GO:0033540) **[see****Supporting Information—Table S4****]**.

Among the 227 common downregulated DEGs in ‘4610’ and Rondo, eight DEGs encoded photosystem proteins **[see****Supporting Information—Table S2****]**. The GO enrichment analysis results showed 7 BP, 5 MF and 13 CC terms **[see****Supporting Information—Table S4****]**. Many of the GO terms were related to photosynthesis, including photosynthesis (GO:0015979), organophosphate metabolic process (GO:0019637), photosystem I (GO:0009522), photosystem (GO:0009521), PSII (GO:0009523), photosynthetic membrane (GO:0034357), thylakoid (GO:0009579), chloroplast thylakoid membrane (GO:0009535) and chloroplast (GO:0009507) **[see****Supporting Information—Table S4****]**.

### Drought-responsive DEGs throughout the two time points of the grain-filling stage

In ‘4610’, there were 925 out of 1107 DEGs that uniquely identified in the early stage of grain filling (14-day time point) compared to the later stage (21-day time point). There were 497 out of 679 DEGs that were uniquely identified in the later stage compared to the earlier one **[see****Supporting Information—Table S5**; **Supporting Information—Fig. S2****]**. On the other hand, Rondo had 707 out of 925 DEGs that uniquely identified in the earlier stage of grain filling compared to the later stage of grain filling; and there were 4881 out of 5099 DEGs that uniquely identified in the later grain-filling stage compared to the earlier stage **[see****Supporting Information—Table S5**; **Supporting Information—Fig. S2****]**.

We further examined DEGs that were common in both time points. For ‘4610’, there were 104 DEGs that were in common, while Rondo had 77 DEGs that commonly downregulated in both time points. Among 104 DEGs, 14 of them had been annotated with drought tolerance or stress response, including bZIP TF, LEA proteins, auxin-responsive protein, RIC2 family protein, GST, Rab protein, NAC-domain proteins and HSPs **[see****Supporting Information—Table S5****]**.

On the other hand, Rondo had 107 upregulated DEGs and 47 downregulated DEGs consistent in both time points, with 12 DEGs were annotated as drought tolerance or stress response **[see****Supporting Information—Table S5****]**. Out of 12 stress-related DEGs, 3 DEGs were cytochrome P450 family proteins (*Os02g0185200*, *Os02g0503900*, *Os03g0760300*). Interestingly, none of the cytochrome P450 family proteins were identified in ‘4610’ in the earlier stage, but there were 6 DEGs of cytochrome P450 family proteins identified in the later stage **[see****Supporting Information—Table S5****]**.

A total of 18 DEGs were consistently upregulated in both time points in both genotypes, with log2 fold-change from 0.58 to 3.89 in earlier stage, and log2 fold-change from 0.5 to 2.97 in later stage. There were 11 DEGs that have been annotated as stress-related functions, one TF, three unknown function DEGs and the other three stress-unrelated DEGs (Table 3).

**Table 3.** Log2 fold-change of DEGs commonly in ‘4610’ and Rondo in both reproductive (Day 14) and grain-filling (Day 21) stages. ‘*’ indicates genes may have stress-related function.

		Day 14		Day 21	
Gene ID	Annotation	‘4610’	Rondo	‘4610’	Rondo
Os01g0200300	Similar to Homeobox-leucine zipper protein HOX29, Homeodomain transcription factor	2.57	1.21	1.22	1.28
Os01g0310100*	Similar to PLDP1 (phospholipase D)	0.80	1.18	0.94	1.42
Os01g0658900*	OSBZ8, bZIP transcription factor	1.32	2.51	1.25	2.52
Os01g0705200*	Late embryogenesis abundant (LEA) protein repeat-containing protein	3.89	3.89	2.36	2.85
Os02g0463401	Conserved hypothetical protein	2.04	0.72	1.05	1.27
Os02g0824500*	Similar to Remorin	2.87	1.51	1.17	1.16
Os03g0277300*	Heat shock protein 70	1.65	1.52	1.05	2.06
Os03g0286900*	RCI2 (rare cold-inducible 2) family protein, Drought resistance	2.65	1.57	2.28	2.97
Os03g0305600*	Mitochondrial import inner membrane translocase, subunit Tim17/22 family protein	2.86	1.55	2.16	2.90
Os03g0723400	Similar to UFG2, endosperm-specific gene 53	3.35	2.20	1.85	2.38
Os05g0373900	Similar to Eukaryotic peptide chain release factor subunit 1 (eRF1)	1.52	1.50	1.17	2.38
Os07g0190800*	Similar to Thioredoxin h	1.01	1.00	1.05	1.07
Os07g0604000*	Similar to 6-phosphogluconolactonase-like protein	1.38	0.86	0.94	1.54
Os08g0403300	Heavy metal transport/detoxification protein domain-containing protein	1.71	2.77	1.50	2.80
Os08g0425800	Conserved protein, expressed	0.71	0.58	0.50	0.78
Os09g0109600*	Small peptide, Drought tolerance	2.76	2.17	1.92	2.61
Os09g0572400*	ABC transporter, ATP-binding protein, putative, expressed	0.60	0.79	0.66	0.93
Os11g0533400	Conserved hypothetical protein	3.07	1.27	1.79	1.61

### Expression patterns of genes involved in signalling cascades and transcriptional control

Several known drought tolerance genes were identified in our study; some were commonly upregulated in both genotypes and time points, including *OsDDR1* (*Os09g0109600*)*, Os02g0824500* (*Remorin*)*, Os01g0310100* (*Phospholipase D P1, PLDP1*) and the well-known drought-tolerant gene rare cold-inducible 2 (*RCI2*) (Li *et al.* 2014) (Fig. 3). *OsDDR1*, which stands for drought and salt stress response 1, is a small peptide protein that can be induced by several environmental stresses, including drought, salinity, ABA and H_2_O_2_ treatment, and has been shown to enhance drought tolerance by overexpression (Cui *et al.* 2018). *Os01g0310100*, annotated as *OsPLDzeta2*, is one of the Phospholipase D (PLD) gene family involved in lipid-mediated signalling. PLD genes can be grouped into several different categories, including alpha, beta, gamma, delta and zeta, according to their structure, and different PLD gene categories also have different functions (Qin and Wang 2002). For example, overexpression of *PLDalpha* was reported to enhance tolerance to drought and osmotic stress in *Arabidopsis* (Wang *et al.* 2014), *PLDbeta* identified as a disease resistant-related gene in rice (Yamaguchi *et al.* 2009) and *PLDzeta1* and *PLDzeta2* had distinct effects on salinity stress response in *Arabidopsis* (Ben Othman *et al.* 2017). *OsPLDzeta2* was reported inducible under drought and cold stress in rice (Singh *et al.* 2012). *OsPLDzeta2* has not yet been functionally annotated; however, based on the function of other PLD groups and the expression profile in the current study (i.e. Rondo had a higher expression level than ‘4610’), we suggest that *OsPLDzeta2* may play a negative role in drought tolerance in rice (Distéfano *et al.* 2015; Ben Othman *et al.* 2017). In this study, *Os02g0824500*, a remorin (REM) similar gene, was first identified in rice under drought stress. It was shown that remorin group 4 (*AtREM4*) genes interact with SnRK1 in *Arabidopsis*, which is known to confer stress tolerance through sugar and hormonal signalling (Cho *et al.* 2012; Lastdrager *et al.* 2014; Son *et al.* 2014). Previous studies had shown that several abiotic stresses, including drought tolerance in tomato and flooding tolerance during germination in rice, could be enhanced by increasing interaction with *SnRK1* (Kretzschmar *et al.* 2015; Yu *et al.* 2018; Yu *et al.* 2021). According to the expression profile, ‘4610’ had relatively higher expression than Rondo in both time points (Fig. 3). This suggests that *Os02g0824500* may also play a role in drought tolerance in rice. On the other hand, a calmodulin-like calcium signalling gene, *OsCML16*, was only increased the expression level in ‘4610’ in 14-day time point. It has been found that *OsCML16* interacts with an ethylene-responsive element-binding factor, *OsERF48*, and enhances root growth and drought tolerance (Jung *et al.* 2017).

We further examined the expression patterns of TFs, known drought-tolerant genes and ROS scavengers. The expression profile showed that a number of TFs were upregulated in both ‘4610’ and Rondo, but most of them had relatively much higher expression in ‘4610’, especially at 14-day time point (Fig. 3). *OsBZ8* (*Os01g0658900*) was the only TF that was differently expressed in both genotypes and in both time points. Comparing to control groups, the log2 fold-change was 1.32 and 1.25 in ‘4610’, and 2.51 and 2.52 in Rondo, in earlier and later grain-filling stages, respectively. *OsBZ8* is a G-box-binding factor-type bZIP protein that binds to ABA-responsive elements (ABREs) such as Rab16 and LEA genes, which regulate the drought stress response gene in the ABA-dependent pathway (Nakagawa *et al.* 1996). Other than *OsBZ8*, there were three other bZIP TFs that were differentially expressed under drought conditions. A TGA-type bZIP TFs (*Os04g0637000*) was exclusively expressed in Rondo. The *Os04g0637000* homologous gene in *Arabidopsis* has been known to play a role in pathogen resistance and root hair development (Wang and Fobert 2013; Canales *et al.* 2017). Interestingly, the expression pattern of the other two bZIP TFs, *Os01g0867300* and *Os02g0766700*, was similar in ‘4610’ and Rondo but at different time points. Both genes are related to ABA signalling under abiotic stress (Amir Hossain *et al.* 2010). However, they were upregulated at earlier time in ‘4610’ and later in Rondo (Fig. 3).

Several TFs shared the same trend that commonly upregulated in ‘4610’ at earlier stage and in Rondo at later stage, such as *Os02g0649300* (*HD-ZIP protein*)*, Os03g0820500* (*Actin depolymerizing factor*) and *Os03g0820300* (*TFIIIA-type zinc finger protein*). Some other TFs commonly increased in ‘4610’ in both time points but only in the later time point in Rondo, including the NAC TFs, *Os11g0126900* and *Os12g0123700*. Both *Os11g0126900* and Os12g0123700 were categorized into NAC group III, namely stress-responsive NAC genes (SNAC) in a previous study (Fang *et al.* 2008). Notably, there was one TF, WRKY 12 (*Os01g0624700*), which was only upregulated at the earlier time point in ‘4610’ and had log2 fold-change over 6, which means the expression was 64 times higher under drought stress (Fig. 3). WRKY family proteins are a class of plant-specific TFs that involve in several stress response pathways (Liu *et al.* 2005; Ryu *et al.* 2006). WRKY family proteins that were reported previously in rice were mainly in pathogen defence regulation. However, several studies revealed that WRKY genes also play a role in enhancing tolerance to abiotic stresses such as drought, cold and salinity in soybean, wheat, *Arabidopsis* and rice (Zhou *et al.* 2008; Wu *et al.* 2009; Niu *et al.* 2012). Taken together, our data suggest that ‘4610’ might have triggered the signalling pathway earlier in response to drought stress than Rondo and had a better response outcome than Rondo accordingly.

Aside from ABA-induced gene families, DREB also regulates drought and cold stress response genes via the ABA-independent pathway (Shinozaki and Yamaguchi-Shinozaki 2000; Lata and Prasad 2011). The current transcription profile has a total of 19 annotated DREB family genes in rice. Our data showed that none of them were identified at the 14-day time point, and only four of them were identified solely at Rondo at the 21-day time point (Fig. 3). In contrast, ‘4610’ had many TFs turned on and off much earlier, including WRKY TF 12 (*Os01g0624700*) and bZIP TFs (*Os01g0867300*, *Os02g0766700*). This profile suggests that the stress-responsive reactions in Rondo had a distinct mechanism from ‘4610’, and the delayed and prolonged signalling might lead to overreacting response detected at the later time point.

### Expression patterns of stress response genes

#### ROS scavengers enhanced drought tolerance.

Reactive oxygen species scavengers play an important role in stress tolerance mechanism in plants (Das and Roychoudhury 2014). Under drought stress, the increased ROS accumulation (Mittler 2002) causes oxidative stress and damage in different levels such as proteins, lipids, DNA and RNA (Sgherri *et al.* 1993; Moran *et al.* 1994; Boo and Jung 1999). There are ROS scavenging mechanisms in plants that help protect the cells under drought stress, such as the scavenging enzymes and non-enzyme antioxidants. Two main enzymes are acting in the ascorbate/glutathione scavenging pathway, the APX and the glutathione reductase (GR), which convert toxic O_2_^−^ to H_2_O (Foyer and Noctor 2011). Other ROS scavenging enzymes include superoxide dismutase (SOD), catalase (CAT), peroxidase (POD) and GSTs (Cruz de Carvalho 2008; Kumar and Trivedi 2018). There were eight ROS scavengers identified in the current study, including mitochondrial sulfur dioxygenase (*OS01G0667200*), peroxidase 11 precursor (*Os06g0274800*), *OsAPx7* (*Os04g0434800*) and GSTs (*Os10g0529500*, *Os01g0692000*, *Os03g0785900*, *Os01g0949750*, *Os10g0528100*) (Fig. 3). Except for the peroxidase 11 precursor, which was solely upregulated in Rondo at the 14-day time point, the rest of ROS scavengers had relatively higher expression in ‘4610’, especially at the 14-day time point. Glutathione-S-transferases were the most abundant ROS scavengers in the current study. Although GST is not directly involved in the ascorbate/glutathione scavenging pathway, a previous study revealed that GST could protect the cell from oxidative damage by quenching reactive molecules with the addition of glutathione (Kumar and Trivedi 2018). Glutathione-S-transferases have also been proven to enhance chilling and drought tolerance in rice (Guo *et al.* 2006). Among the five GSTs identified, four of them were upregulated during earlier grain-filling stage in ‘4610’ and none were upregulated during this earlier stage in Rondo. Notably, our study did not identify any significant DEGs belong to the SOD and CAT families. These data again showed that most ROS scavengers responded much earlier and more upregulated in ‘4610’ than in Rondo. These ROS scavengers removed the excess ROS, relieve oxidative stress and subsequently prevent further damage. This factor may also partially contribute to the higher tolerance of ‘4610’ under drought than Rondo.

#### Chaperons induced under drought stress.

We investigated the expression pattern of LEA genes, directly downstream genes of bZIP TFs. Late embryogenesis abundant protein helps offset the harmful effects of water deficit by preventing protein aggregation in the cell during water stress that allows the cell to maintain its function (Goyal *et al.* 2005). Therefore, LEA proteins were annotated as drought and cold tolerance proteins and have been overexpressed in various plants, including brassica and rice, to improve their tolerance to such stresses (Xiao *et al.* 2007; Dalal *et al.* 2009). In our study, eight LEA genes were differentially expressed; however, only one, i.e. *Os01g0705200*, was increased in both ‘4610’ and Rondo at the two time points. On the other hand, three of them were upregulated in ‘4610’ at the earlier stage but in Rondo at the later stage, and four LEA genes were solely increased in Rondo at the later stage (Fig. 3). Our results clearly showed that at the earlier stage, LEA family proteins relatively had higher expression levels in ‘4610’ than Rondo. This result concurred with the expression pattern of TFs that has been discussed above, which suggests that ‘4610’ responds to the signalling cascades earlier in the grain-filling stage than Rondo, which may partially contribute to better drought tolerance.

#### Other genes contribute to drought tolerance.

Several cytochrome p450 genes were upregulated in both stages in Rondo (Fig. 3). This may be the unique drought response strategy in Rondo. Previous studies reported that the expression of cytochrome p450 genes improves drought tolerance by fine-tuning GA-to-ABA balance and mediating metabolic responses (Nam *et al.* 2014; Tamiru *et al.* 2015) and grain formation during drought stress (Pandian *et al.* 2020). Several of these genes were upregulated in both time points of grain filling, some during the earlier stage, while others during the later stage. It was unexpected that these gene families were upregulated in Rondo, the more susceptible cultivar to drought. However, it is possible that the timing of the gene expression plays a significant role (most of the drought transcriptomic studies were performed during reproductive stage) and other gene networks could possibly mask the significant effects of the gene families. This phenomenon needs further investigation. However, at the same time, this also offers some opportunities for future molecular genetic manipulation to enhance drought tolerance of Rondo via modification of the key cytochrome p450 gene families.

Other than consistently expressed DEGs, there were several DEGs that were upregulated in ‘4610’ at the earlier stage but not in Rondo at the same stage (Fig. 3), including two-pore potassium channel, *OsTPKb* (*Os07g0108800*), Rab21 (*Os11g0454300*), Rab16 (*Os11g0454000*), *OsIAA6* (*Os01g741900*), an auxin-responsive protein, and beta-carotene hydrolase (*Os03g125100*). Vacuolar two-pore potassium channels are important for the regulation of cellular potassium levels. Previous studies have shown that potassium uptake was greater in *OsTPK*-overexpressed rice lines. With higher cytoplasm to vacuole potassium ratio, plants tend to have better osmotic and drought tolerance (Ahmad *et al.* 2016); on the contrary, *OsTPK* knockout mutants showed lower drought tolerance (Ahmad *et al.* 2016; Chen *et al.* 2017). Both Rab16 and Rab21 are ABA-responsive genes, which can sense the existence of ABA trigger downstream stress response signalling. In addition, Rab21 was also known to increase rapidly after drought stress throughout all rice growth stages (Skriver *et al.* 1991; Yi *et al.* 2010). Rab proteins, the small G protein family, involved in different activities, including vesicle trafficking, intracellular signalling events, various physiological processes and stress response (Choudhary and Padaria 2015). The link between auxin expression and drought responses has been reported in a previous study with an example of *OsIAA6*. The study showed that *OsIAA6*-overexpressed rice had better drought tolerance, and it may be due to the control of tiller outgrowth (Jung *et al.* 2015). *OsIAA* and another gene, *OsDHODH*, which encodes cytosolic dihydroorotate dehydrogenase (DHODH) (Liu *et al.* 2009) were upregulated in both stages. *OsDHODH* is involved in plant stress response, including under salinity or drought stress conditions (Liu *et al.* 2009). Beta-carotene hydrolase is known to be involved in the synthesis of zeaxanthin, a carotenoid precursor of ABA biosynthesis. It has been reported that rice had significantly increased drought and oxidative stress tolerance with a higher level of zeaxanthin and ABA level (Du *et al.* 2010). Again, these data also suggest that ‘4610’ had a more rapid and better response against drought stress than Rondo.

### Yield-related performance under drought stress and its closely related gene regulation

In agreement with previous studies, our results also showed that filled grain number, spikelet fertility, grain weight, plant dry weight, panicle length and yield were decreased under drought stress (Garrity and O’Toole 1994; van der Weijde *et al.* 2017; Zhang *et al.* 2018). Based on the DEGs and enrichment analysis, we identify several genes common in ‘4610’ and Rondo that may partially contribute to the phenotypic differences. For example, *Os01g0322700*, annotated as regulator of panicle-branching, grain weight, grain yield and photosynthesis; *GW5* (*Os05g0158500*), a serine carboxypeptidase, which acts as a positive regulator of grain size; cytokinin-activating enzyme (*Os01g0588900*) and cytokinin signalling kinase (*Os02g0738400*) were downregulated in both ‘4610’ and Rondo in the 14-day time point but not in the 21-day time point **[see****Supporting Information—Table S1****]**. Conversely, during the later grain-filling stage, there were no common downregulated DEGs across the two genotypes that directly related to agronomic traits, but there were eight downregulated DEGs that encoded photosystem proteins (*Os01g0773700*, *Os03g0747700*, *Os03g0778100*, *Os07g0148900*, *Os07g0673550*, *Os08g0119800*, *Os09g0475800* and *Os09g0481200*) **[see****Supporting Information—Table S2****]**. Photosynthesis provides a carbon source for starch accumulation during the grain-filling stage in cereals (Prathap *et al.* 2019). Therefore, a decreased photosynthesis may contribute to poor grain filling, which affects the size and quality of the rice grains. In addition, some grain and yield-related genes were downregulated in Rondo, including regulation of grain size (*Os06g0130400*, *Os09g0517600*, *Os12g0610200*, *Os04g0645100*), regulation of grain shape (*Os09g0448500*) and regulation of yield (*Os01g0878400*, *Os07g0603800*) **[see****Supporting Information—Table S2****]**. Therefore, our study suggests that the inferiority of agronomic trait parameters under drought stress conditions might be partially contributed by the downregulation of both groups of trait-related genes and photosynthesis-related genes.

### Potential novel drought-responsive genes

To explore the potential novel drought-responsive genes, we examined the DEGs without functional annotation. During the earlier time point, 59 DEGs annotated as ‘conserved hypothetical gene’, 45 DEGs annotated as ‘hypothetical gene/protein’ and 23 DEGs annotated as ‘domain of unknown function (DUF) proteins’ in ‘4610’; on the other hand, 133 DEGs annotated as ‘conserved hypothetical gene’, 34 DEGs annotated as ‘hypothetical gene/protein’ and 15 DEGs annotated as ‘DUF proteins’ in Rondo **[see****Supporting Information—Table S1****]**. During the later stage, 29 DEGs annotated as ‘conserved hypothetical gene’, 25 DEGs annotated as ‘hypothetical gene/protein’ and 6 DEGs annotated as ‘DUF proteins’ in ‘4610’; on the other hand, 326 DEGs annotated as ‘conserved hypothetical gene’, 168 DEGs annotated as ‘hypothetical gene/protein’ and 107 DEGs annotated as ‘DUF proteins’ in Rondo **[see****Supporting Information—Table S2****]**. To narrow down our potential novel gene pool, three screening criteria were developed: (i) DEGs had log2 fold-change larger than 1 and consistently upregulated in both ‘4610’ and Rondo in the same stage, (ii) DEGs in only one genotype but consistently upregulated in both stages with log2 fold-change larger than 1 and (iii) DEGs consistently in both genotypes and both stages. A total of 36 DEGs met our criteria with log2 fold-change ranged from 0.72 to 7.54 (Table 4). Two DEGs that were upregulated in all four groups (Os02g0463401 and Os11g0533400), and in the case of these two genes, the log2 fold-change in ‘4610’ during earlier stage was the highest (2.04 and 3.07, respectively). Four DEGs were upregulated only in ‘4610’ during both stages (Os01g0184050, Os02g0259900, Os03g0267100 and Os08g0110600). There were also several DEGs that were upregulated in both genotypes during grain filling, and one of the genes with the largest log2 fold-change was Os02g0609000 (6.04 and 7.54 in ‘4610’ and Rondo, respectively). Some of these genes may have functions related to drought stress tolerance; however, this requires further investigation.

**Table 4.** Log2 fold-change of novel drought-responsive upregulated DEGs.

		Reproductive stage		Grain-filling stage	
Gene ID	Annotation	‘4610’	Rondo	‘4610’	Rondo
Os01g0128250	Hypothetical gene	ns	ns	2.35	3.79
Os01g0184050	Hypothetical protein	1.34	ns	1.62	ns
Os01g0200350	Hypothetical protein	2.36	ns	1.51	1.84
Os01g0214500	Conserved hypothetical protein	1.66	ns	1.07	2.48
Os01g0229600	Conserved hypothetical protein	1.77	1.18	ns	ns
Os01g0652375	Hypothetical protein	ns	ns	1.61	2.57
Os01g0652450	Hypothetical gene	ns	5.46	ns	2.26
Os01g0727700	Hypothetical conserved gene	1.92	ns	2.30	4.06
Os01g0727820	Hypothetical protein	ns	1.78	ns	1.04
Os01g0838350	Conserved hypothetical protein	ns	ns	1.12	1.82
Os01g0888900	Conserved hypothetical protein	ns	ns	2.00	3.05
Os02g0140800	Conserved hypothetical protein	2.12	1.28	ns	1.47
Os02g0258800	Conserved hypothetical protein	1.77	2.29	ns	ns
Os02g0259900	Conserved hypothetical protein	1.04	ns	1.64	ns
Os02g0463401	Conserved hypothetical protein	2.04	0.72	1.05	1.27
Os02g0463401	Conserved hypothetical protein	ns	ns	1.05	1.27
Os02g0514326	Hypothetical protein	ns	ns	2.04	2.41
Os02g0609000	Hypothetical protein	ns	ns	6.01	7.54
Os02g0740500	Conserved hypothetical protein	ns	2.09	ns	1.7
Os03g0257700	Hypothetical protein	ns	2.43	ns	1.5
Os03g0267100	Hypothetical protein	1.2	ns	1.08	ns
Os03g0305550	Hypothetical gene	2.45	ns	2.43	3.68
Os03g0381500	Conserved hypothetical protein	1.96	1.36	ns	ns
Os03g0809400	Hypothetical conserved gene	ns	ns	1.17	1.11
Os05g0299500	Protein of unknown function DUF914, eukaryotic family protein	ns	ns	1.23	1.55
Os05g0390550	Conserved hypothetical protein	1.92	2.71	ns	3.63
Os06g0651200	Conserved hypothetical protein	2.26	ns	1.18	1.09
Os06g0700550	Hypothetical protein			1.22	2.30
Os07g0564200	Conserved hypothetical protein	1.07	1.9	ns	1.16
Os08g0110600	Protein of unknown function DUF1442 domain-containing protein	2.27	ns	1.25	ns
Os08g0286500	Hypothetical conserved gene	ns	ns	1.74	2.15
Os09g0425400	Hypothetical protein	1.9	ns	1.29	1.77
Os09g0426000	Protein of unknown function DUF6, transmembrane domain-containing protein	ns	ns	1.65	2.65
Os11g0533400	Conserved hypothetical protein	3.07	1.27	1.79	1.61

## Conclusions

This transcriptomics study showed the complexity of the drought response mechanism in rice during grain filling. Phenotypic and transcriptomic data showed that both ‘4610’ and Rondo were affected by drought stress. Both the moderate-tolerant and susceptible genotypes had variable responses against drought stress, including drought sensing, signalling, downstream regulation and ROS scavenging. By comparing the expression of these key genes, we suggest that ‘4610’ was less affected by drought stress due to its more rapid stress response and higher expression level of key drought-tolerant genes, LEA proteins, ROS scavengers, APXs and GSTs. Some of these genes can be potential targets for further study and manipulation to develop more resilient high-yielding rice under drought stress conditions.

## Supporting Information

The following additional information is available in the online version of this article—

**Table S1.** Differentially expressed genes at the earlier grain-filling stage (Day 14).

**Table S2.** Differentially expressed genes at the later grain-filling stage (Day 21).

**Table S3.** Gene ontology at the earlier grain-filling stage (Day 14).

**Table S4.** Gene ontology at the later grain-filling stage (Day 21).

**Table S5.** Differentially expressed genes at both time points of the grain-filling stage (Day 14 and Day 21).

**Figure S1.** Principal component analysis (PCA) of RNA-seq samples separated by the two time points (Day 14 vs. Day 21).

**Figure S2.** Venn diagrams of differentially expressed genes (DEGs) for each genotype.

plab043_suppl_Supplementary_Figures

plab043_suppl_Supplementary_Table_S1

plab043_suppl_Supplementary_Table_S2

plab043_suppl_Supplementary_Table_S3

plab043_suppl_Supplementary_Table_S4

plab043_suppl_Supplementary_Table_S5

## Data Availability

Data are available as **Supporting Information**.

## Notes

**Phenome, Genome & Environment. Chief Editor:** Colleen Doherty
