# Supplementary material for: Transcriptome profiling of two rice genotypes under mild field drought stress during grain-filling stage
Source: AoB Plants. 2021 Jul 5;13(4):plab043. doi: 10.1093/aobpla/plab043 (PMC8331054; doi:10.1093/aobpla/plab043)
Supplement: plab043_suppl_Supplementary_Figures [file plab043_suppl_supplementary_figures.pdf]

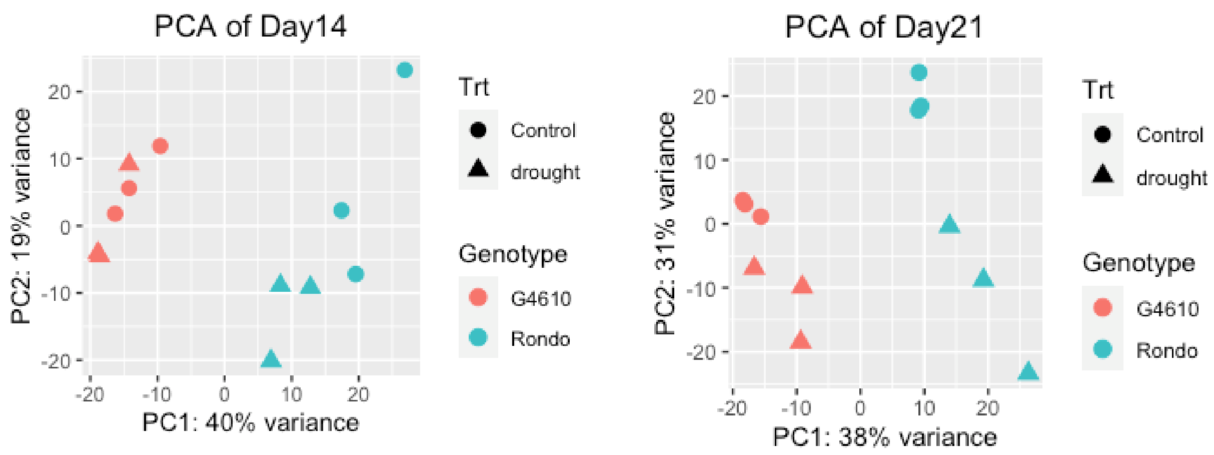

Supplementary Figure 1. Principal component analysis (PCA) of RNA-seq samples separated by the two time points (Day 14 vs. Day 21). The color differences indicate the different genotypes ('4610 vs. Rondo), and the shape differences indicate the different treatments (control vs. drought).

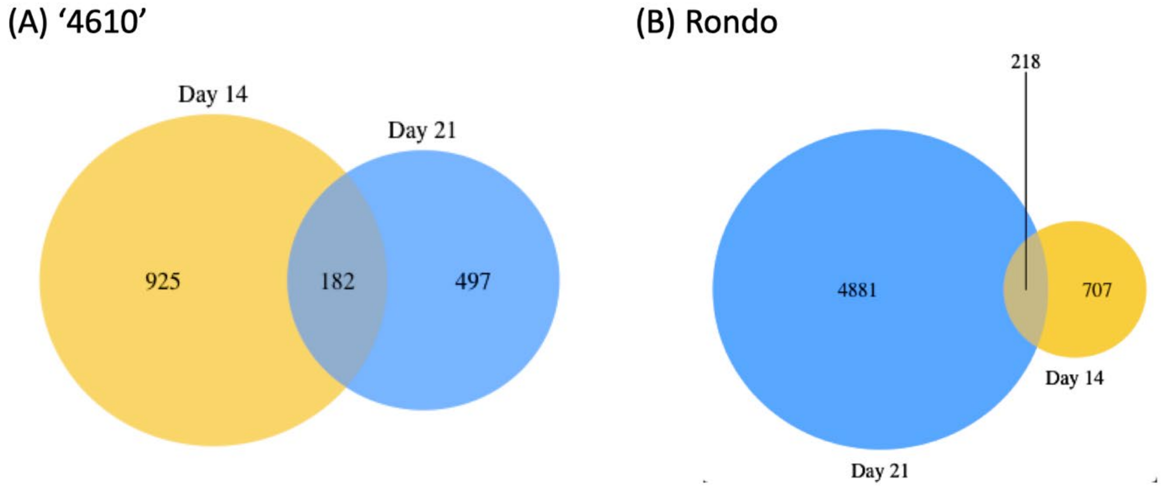

Supplementary Figure 2. Venn diagrams of differentially expressed gene (DEGs) for each genotype, (A) '4610' and (B) Rondo, between early grain-filling stage (Day 14) and late grain-filling stage (Day 21).
